# Typological differentiation and influence mechanisms of gender role attitudes among Chinese women

**DOI:** 10.3389/fpsyg.2025.1547390

**Published:** 2025-04-01

**Authors:** Ruihua Chi, Jin Zhou

**Affiliations:** School of Social Science, Soochow University, Suzhou, China

**Keywords:** Chinese women, gender role attitudes, gender equality, typological differentiation, modernity theory

## Abstract

With the current accelerated evolution of globalization, developing countries, represented by China, are experiencing a reshaping of gender concepts due to the impact of multiculturalism. Based on data from the China General Social Survey 2010–2021, this study uses latent profile analysis to identify different types of Chinese women’s gender role attitudes. In addition, this study comprehensively analyzes the influencing mechanisms of the differentiation of gender role attitudes types, and longitudinally compares the evolutionary trends of Chinese women’s gender role attitudes types. The conclusions of the study are as follows. First, Chinese women’s gender role attitudes are diversified, and there are four types: multidimensional traditional, social division of labor traditional, innate ability traditional and completely modern. Secondly, according to the different influencing mechanisms, this paper classifies the factors affecting the differentiation of Chinese women’s gender role attitudes into three categories: external environment-driven, intrinsic interest-driven, and comprehensive-driven. Finally, since 2010, the pattern of Chinese women’s gender role attitudes has evolved from being dominated by the “multidimensional traditional type” to the “fully modern type.” However, through precise characterization, it is found that the evolutionary trends of women holding the four types of gender role attitudes are different.

## 1 Introduction

Over the past two decades, China has made considerable efforts to achieve gender equality by addressing the gender gap in education, safeguarding women’s rights, and providing more employment opportunities for women (Li, 2021). However, many studies indicate that the pursuit of gender equality in China has encountered significant challenges. Particularly during the first decade of the 21st century, traditional gender concepts have made a notable resurgence. Research shows that from 2000 to 2010, women’s gender role attitudes in China regressed toward traditional norms, characterized by the belief that “men should be the breadwinners while women should manage the household” and that “doing well is not as favorable as marrying well” (Yang et al., 2014). Gender role attitudes serve as a crucial indicator for assessing the extent of gender equality achieved (Liu and Tong, 2014). Following the issuance of the “Outline for the Development of Chinese Women (2011–2020),” China’s gender equality initiatives have entered a new phase. The macro environment within China has undergone significant transformation, with economic development shifting from a stage of rapid growth to one focused on high-quality progress, and urbanization levels continuing to rise. Against this social backdrop, the critical question this study addresses whether the gender role attitudes of Chinese women continue to reflect the traditional trends of the first decade of the 21st century or whether they will progress toward modernization in the second decade.

Gender role attitudes reflect how individuals perceive the social roles of men and women, with a central focus on rights and gender norms (Li et al., 2024; Wang and Wang, 2019). Numerous empirical analyses have been conducted regarding the dimensions and influencing factors of gender role attitudes (Thornton and Freedman, 1979). However, existing research primarily adopts a “variable-centered” paradigm, emphasizing causal relationships between gender role attitudes and other variables while neglecting the internal structural differentiation of these attitudes (Jan and Janssens, 1998). Causal mechanism analysis centered on variables focuses on revealing the “average” effect of gender role attitudes, which evidently does not align with the realities of the situation (Abeya, 2015). On the one hand, gender role attitudes encompass a complex framework that includes aspects such as gendered division of labor, gender capabilities, romantic preferences, and household responsibilities (Yu and Chen, 2021). As China navigates a critical period of transformation, it is possible that women may experience inconsistencies in their identification with various dimensions of gender role attitudes. On the other hand, the diversity within China’s female population, including differences in age, income, urban–rural status, and educational attainment, may result in varying perceptions among women regarding multiple aspects of gender role attitudes. Based on this, this study will focus on addressing the following three research questions. First, to identify the potential types of gender role attitudes among Chinese women and their characteristics. Second, to examine the temporal evolution patterns of different types of gender role attitudes. Third, to explore the factors influencing the differentiation of gender role attitudes among Chinese women.

Therefore, this study employs a “person-centered” latent profile analysis (LPA) method (Gabriel et al., 2018), utilizing multi-point data from the China General Social Survey, to explore the different types of gender role attitudes among Chinese women. Additionally, it employs multinomial logistic regression to analyze the impact of various internal and external factors on these diverse types of gender role attitudes (Abeya, 2015). Ultimately, the study aims to provide a longitudinal comparison and characterization of the evolving trends in gender role attitudes among Chinese women, with the hope of offering valuable insights for the advancement of global gender equality initiatives.

## 2 Literature review

### 2.1 Concept and structure of gender role attitudes

Early studies, influenced by biology, argued that gender differences stem from physiological characteristics, with the most representative theory being biological determinism (Miller and Costello, 2001). This theory posits that physiological differences determine the distinct temperament traits and social division of labor between men and women. However, with the emergence of social gender theory, scholars recognized that gender role attitudes were co-constructed by institutional structures, cultural norms, and individual practices (Sun, 2018). This theory emphasizes that the division of labor between the sexes is a result of the reproduction of traditional gender culture, with the core mechanism being the symbolic transformation of physiological differences into a legitimate basis for social hierarchy (Oakley, 2016). This theoretical shift reveals that gender role attitudes are a multidimensional dynamic system with complex interactions between various dimensions.

Existing research deconstructs gender role attitudes through five dimensions: social gender division of labor, gender capabilities, career and marriage choices, social competitiveness, and domestic labor division (Feng and Xiao, 2014). These dimensions are interrelated and form a network for the reproduction of gender power relations. The social division of labor shapes gender cognitive frameworks through the public-private domain divide, while occupational gender segregation and family role disciplining solidify traditional division patterns, weakening women’s demands for equal rights in the public sphere (Xiong et al., 2018). At the level of cultural norms, gender capabilities are preconfigured and reproduced through educational screening and career evaluation narratives, reinforcing hierarchical orders of capability, and linking with domestic labor practices (Qin, 2019). Career and marriage choices are constrained by the legitimacy of the division of labor; when this legitimacy is strengthened, women’s endorsement of the notion “a good marriage is better than a good career” increases (Li and Wang, 2021). Domestic labor division reflects the outcome of gender division and cultural norm enactment, with feminine roles reinforcing the “woman’s place in the home” ideology, weakening competitiveness demands, and forming a feedback loop of reinforcement (Liu and Tong, 2014; Xiong et al., 2018).

The hierarchical relationships between these dimensions highlight the dual dilemmas of traditional research paradigms: First, measurement methods exhibit cognitive fragmentation, with existing research generally adopting a homogenized approach that overlooks the asymmetric interactions between dimensions (Feng and Xiao, 2014). Second, they neglect contradictory cognitions, where some women hold modern stances in the public sphere but continue traditional practices in the private sphere. Homogenized measurements obscure the internal tensions in gender role attitudes.

### 2.2 Influencing factors of women’s gender role attitudes

There are two primary perspectives on the factors influencing gender role attitudes and their mechanisms: external environment determinism and internal interest determinism (Zhu and Li, 2015). The former posits that gender role attitudes are predominantly shaped by the environment, with individual behaviors and decisions as responses to external conditions. The latter emphasizes the critical role of individual consciousness, intention, and agency in shaping gender role attitudes. However, some scholars argue that the formation of gender role attitudes results from the interplay between external environment and internal interests (Xu et al., 2010).

This study integrates these two theories, identifying 10 factors influencing women’s gender role attitudes: parents’ education levels, marital status, age, urban–rural differences, employment status, individual education level, political affiliation, personal income, and subjective class identity. It also delves into the mechanisms of these factors within the Chinese context.

First, the external environment determinism in China exhibits institutional deformation and spatial heterogeneity. The urban–rural dual structure imposes triple constraints on rural women: unequal access to education, limited employment opportunities, and insufficient social security. Urban women, on the other hand, face the paradox of modernity: while higher education fosters the growth of “fully modern” groups, market risks compel highly educated women to achieve class reproduction through “strategic traditionalization” (e.g., accepting bride prices) (Fan and Marini, 2000). Intergenerational transmission within families also reveals gendered differentiation: fathers’ education reinforces instrumental rationality through professional networks, while mothers’ education mitigates traditional views on marriage and romance through emotional interaction (Dotti Sani and Quaranta, 2017; Jan and Janssens, 1998).

Second, the internal interest determinism requires adaptive reconstruction within China’s family-based ethics. Studies show that nearly half of high-income women still hold the belief that marrying well is better than working well, indicating that individual rationality is subsumed under the logic of maximizing family interests (Li and Wang, 2021). The anti-modernity of subjective class identity highlights cultural constraints: a higher proportion of middle-class women embrace the “virtuous wife and good mother” role compared to lower-class groups (Stickney and Konrad, 2007; Valentova, 2013). Educational empowerment presents a threshold effect: among those with less than a college degree, modernity scores significantly increase with years of education, while for those with a bachelor’s degree or higher, due to “diminishing returns on education” and “intensified motherhood penalty,” the marginal effect of education approaches zero (Bryant, 2003).

The Chinese experience offers a dual critique of traditional theories. External environment determinism overlooks the dynamic impact of state governance tools such as birth policies, while internal interest determinism underestimates the shaping power of the “family-state isomorphism” ethic. By integrating diverse factors, this study not only maintains the core propositions of classical theories but also, through localized adaptation, contributes a new analytical framework to global gender studies.

### 2.3 The evolution of women’s gender role attitudes

This study adopts modernity theory as its core analytical framework, exploring how industrialization, urbanization, and the expansion of education drive the modernization of gender role attitudes (Thornton and Young-DeMarco, 2001). Cross-national studies reveal a steady trend toward gender equality in Western countries since the 1970s, with the core mechanism being the de-gendering of the labor market and the spread of individualistic values, gradually undermining the legitimacy of traditional gender divisions (Liu and Tong, 2014; Twenge, 1997; Valentova, 2013).

However, the linear progress narrative assumed by modernization theory encounters adaptive transformations of indigenous cultural genes when explaining changes in China’s gender role attitudes. Data indicate that while rising female education levels and labor participation rates partially support this theory, the cultural lag effect highlighted by social and cultural theories significantly constrains the homogeneity of modernization processes (Wu et al., 2022). Confucian ethics, through the “family-state isomorphism” network of meanings, perpetuate the “men work outside, women manage the home” norm within kinship systems. This cultural inertia manifests in the marriage market as a collusion between instrumental rationality and traditional ethics—many highly educated women still prioritize marriage as a strategy for social mobility (Yang et al., 2014). Industrialization and education expansion have led to substantial progress in gender equality in the public sphere, yet the resurgence of tradition in the private sphere fundamentally challenges the modernity paradigm (Liu and Tong, 2014; Yang et al., 2014). This contradiction is essentially a result of the interplay between cultural autonomy and institutional complexity.

Gender role socialization theory further explains the enduring influence of institutional forces. The one-child policy, while promoting educational investment in women, also reproduces traditional order under the guise of modernization through media-driven “leftover women” stigma and familial transmission of marriage anxiety (Xu, 2016). The tension between progress and conservatism finds a micro-mechanism explanation through social learning theory: as market economic risks intensify, young women’s approval of the notion “a good marriage is better than a good job” increases (Li and Wang, 2021), reflecting a strategic invocation of traditional gender protection mechanisms by observing maternal experiences (e.g., workplace discrimination) and evaluating behavioral consequences (e.g., opportunity costs of marriage and childbirth) (Bussey and Bandura, 1999).

The Chinese case demonstrates that changes in gender role attitudes are not a unidirectional diffusion of modernity but rather a non-equilibrium mosaic formed amid the triple tensions of state governance logic, market risk transfer, and cultural gene activation (Yang et al., 2014). Thus, the evolution of China’s gender role attitudes is essentially a dynamic contest between the push of modernity and the pull of tradition: industrialization and educational expansion provide the structural impetus for modernization, while cultural inertia, institutional shortcomings, and risk aversion create countervailing forces through socialization mechanisms, learning processes, and cultural symbol systems. This model of uneven transformation not only upholds modernity theory’s explanatory power over macro trends but also, through the embedding of local theories, reveals its theoretical blind spots regarding cultural particularities.

## 3 Research design

### 3.1 Data sources

This study utilizes data from the “China General Social Survey (CGSS)” conducted in 2010, 2013, 2015, 2017, and 2021. This social survey is jointly executed by Renmin University of China in collaboration with various academic institutions nationwide. The survey employed a multi-stage stratified sampling method to ensure the randomness of the sample. Since 2003, it has conducted 15 rounds of surveys among urban and rural residents aged 18 and above in 31 provinces (autonomous regions and municipalities) across mainland China, reaching a total of 160,000 respondents. This approach systematically and comprehensively collected data at multiple levels, including individual, family, community, and society. The limitation of CGSS lies in the irregular intervals between survey waves, leading to missing data in certain years. However, CGSS data remains highly representative in China, providing a reliable data source for this study. This study focuses on the gender role attitudes of the female population; thus, male samples and those with missing values in related variables were excluded. The sample sizes for CGSS 2010, 2013, 2015, 2017, and 2021 are 5,018, 4,880, 4,645, 6,149, and 3,234, totaling 23,926.

### 3.2 Variables and measurements

The core variable of this study is gender role attitudes. In the data from CGSS 2010, CGSS 2013, CGSS 2015, CGSS 2017, and CGSS 2021, five items are employed to measure these attitudes. Item A421, “Men prioritize career while women prioritize family,” assesses women’s views on gender division of labor; A422, “Men are inherently more capable than women,” evaluates women’s perceptions of gender abilities; A423, “Doing well is not as important as marrying well,” gauges women’s romantic inclinations and their level of dependence on men; A424, “In times of economic downturn, women employees should be laid off first,” measures women’s attitudes toward competition in the labor market; and A425, “Husbands and wives should share household chores equally,” examines women’s perspectives on domestic labor division. All five items utilize a five-point Likert scale, ranging from 1 (strongly disagree) to 5 (strongly agree). For the first four items, higher values indicate more traditional views among women, while for the fifth item, higher values reflect more modern attitudes; thus, the responses to the fifth item are reverse coded to ensure consistency in the direction of the attitudes measured. This study analyzed the reliability and validity of five indicators of gender role attitudes. The KMO values for the 5 years of data ranged from 0.71 to 0.77 and the Cronbach’s alpha ranged from 0.61 to 0.70, with significance less than 0.001, which suggests that the data from all the years have good reliability and validity.

To facilitate the analysis of the influencing factors model, the independent variables have also been recoded. The analysis utilizes the most recent CGSS data from 2021, thus coding adheres to the latest standards. The independent variables include age, marital status, education level, personal income, political affiliation, urban or rural residency, subjective class identification, employment status, and parental education level. Detailed coding methods and results for all measured variables can be found in Table 1.

**Table 1:** Coding results for measured variables.

Variable	Encoding
Age	Age = year of survey - year of birth. The results were coded as follows: 18–44 years as young = 1; 45–59 years as middle-aged = 2; and over 60 years as old = 3. (Refer to World Health Organization standards).
Marital status	Coding unmarried and cohabiting as unmarried females = 1; coding married, divorced and widowed as females with marital experience = 2.
Individual’s educational level	Higher education level = 1 for university colleges and above; secondary education level = 2 for high schools, junior colleges and technical schools; primary education level = 3 for junior high schools and below.
Annual personal income	Above RMB 80,294 is high income level = 1; RMB 7,869–80,294 is middle income level = 2; below RMB 7,869 is low income level = 3. (Refer to the 2020 National Bureau of Statistics of China standards).
Political profile	People with political beliefs = 1; the masses = 2.
Registered residence	Code according to household registration status: non-agricultural household registration for urban women = 1; agricultural household registration for rural women = 2.
Subjective class identity	For the current subjective class identification, assign 7–10 for upper-class women = 1; 5–6 for middle-class women = 2; 1–4 for lower-class women = 3.
Employment status	Currently employed in non-agricultural work = 1; currently engaged in farming = 2; currently unemployed = 3.
Parents’ education	Parents with a certain level of education = 1; parents with no education = 2.
Gender role attitudes	Retain the original values for items A421, A422, A423, and A424, with a scale of 1–5 indicating the respondent’s level of agreement from “strongly disagree” to “strongly agree.” However, for item A425, since its direction of agreement is clearly opposite to the previous four items, reverse-code its responses.

### 3.3 Data analysis methods

#### 3.3.1 Latent profile analysis

Latent class modeling is a research method that centers on individuals, classifying them by analyzing the probability distribution of their responses to manifest variables. During the classification process, latent class models seek the optimal model by incrementally increasing the number of categories. Latent class modeling is divided into latent class analysis, which uses categorical variables, and latent profile analysis, which uses continuous variables. As gender role attitudes are a continuous variable, this study employs latent profile analysis to identify the categories of gender role attitudes among Chinese women and conducts a longitudinal comparison to examine whether their types and proportions have changed (Gabriel et al., 2018). The fitting process begins with a two-class model, progressively increasing the number of classes, and selecting the optimal model by comparing the fit indices. Commonly referenced indicators include AIC, BIC, aBIC, and Entropy. Among these, smaller values of AIC, BIC, and aBIC indicate better model fit, while a larger Entropy value suggests superior model performance. Furthermore, a category probability must exceed 5% for the classification to be deemed significant.

#### 3.3.2 Logistic regression analysis

The logistic regression model is a type of generalized linear regression model and unordered multicategorical logistic regression model is a model that analyzes the dependent variable with more than two unordered dependent variables (Lubke and Muthén, 2007). In this study, based on the results of the latent class analysis, the dependent variable, gender role attitudes, can be categorized into multiple latent types that are unordered. An unordered multinomial logistic regression model is well-suited for analyzing and fitting the dependent variable. Thus, this study employs an unordered multinomial logistic regression model. The independent variables are incorporated into the model according to the influencing factors analysis framework for the examination of their effects.

## 4 Research findings

### 4.1 Identification of types of gender role attitudes among women

Typological analysis is an important research method in sociology for classifying phenomena, viewed through the lenses of holism and historicism. It examines the actual state of human communal life and compares the characteristics and distinctions of each type, allowing for a clearer understanding of the internal differences among phenomena (Zhang, 2019). This study integrates typological analysis with the characteristics of gender role attitudes among Chinese women, categorizing these attitudes based on the two dimensions of “traditional” and “modern” within the theoretical framework. The internal structure of the Chinese female population is complex, and there may be certain variations in their gender role attitudes across different measurement dimensions. To more accurately describe the current state of gender role attitudes among Chinese women and to precisely characterize their evolutionary trends, this study seeks to identify the existing types within this framework.

Gender role attitudes are treated as continuous variables; thus, latent profile analysis (LPA) using Mplus 8.3 software is employed to identify their types. To enhance the accuracy of characterizing these attitudes, the study utilizes several years of survey data for the analysis of fitting results. Existing research on the evolutionary trends of gender role attitudes primarily relies on survey data prior to 2010, with a lack of focus on data from after that year. Therefore, this study analyzes the China General Social Survey data from 2010 to 2021. To address the representativeness of the sample years, the study selects survey data from the years 2010, 2013, 2015, 2017, and 2021, performing multiple fittings for each year, with the fitting results presented in Table 2.

**Table 2:** Latent category model fit indices for different years.

Year of data	Categories	Npar	AIC	BIC	aBIC	Entropy	LMR-P	Categorical probability
CGSS2010 (*N* = 5,018)	2	16	75,107	75,211	75,161	0.907	0.00	0.30/0.70
	3	22	73,841	73,985	73,915	0.868	0.00	0.29/0.27/0.44
	**4**	**28**	**72,644**	**72,827**	**72,738**	**0.916**	**0.00**	**0.23/0.41/0.28/0.08**
	5	34	71,803	72,025	71,917	0.895	0.00	0.24/0.04/0.24/0.16/0.32
	6	40	71,100	71,421	71,293	0.937	0.00	0.18/0.36/0.03/0.09/0.21/0.13
CGSS2013 (*N* = 4,880)	2	16	69,189	69,293	69,242	0.907	0.00	0.33/0.67
	3	22	68,159	68,302	68,232	0.859	0.00	0.28/0.21/0.51
	**4**	**28**	**67,036**	**67,218**	**67,129**	**0.889**	**0.00**	**0.24/0.41/0.23/0.11**
	5	34	66,579	66,800	66,692	0.887	0.00	0.24/0.10/0.21/0.05/0.39
	6	40	65,740	66,000	65,873	0.858	0.00	0.17/0.24/0.12/0.05/0.32/0.10
CGSS2015 (*N* = 4,645)	2	16	66,022	66,125	66,074	0.923	0.00	0.37/0.63
	3	22	65,104	65,246	65,176	0.852	0.00	0.36/0.47/0.17
	**4**	**28**	**64,056**	**64,237**	**64,148**	**0.905**	**0.00**	**0.40/0.05/0.29/0.26**
	5	34	631,494	63,413	63,305	0.900	0.00	0.30/0.05/0.16/0.09/0.40
	6	40	62,227	62,484	62,357	0.928	0.00	0.07/0.04/0.26/0.15/0.38/0.10
CGSS2017 (*N* = 6,149)	2	16	89,601	89,709	89,658	0.941	0.00	0.44/0.56
	3	22	88,700	88,848	88,779	0.858	0.00	0.43/0.43/0.14
	**4**	**28**	**86,901**	**87,090**	**87,001**	**0.914**	**0.00**	**0.11/0.34/0.36/0.19**
	5	34	85,915	86,144	86,036	0.903	0.00	0.12/0.16/0.29/0.06/0.37
	6	40	84,348	84,617	84,490	0.909	0.00	0.12/0.06/0.27/0.08/0.33/0.14
CGSS2021 (*N* = 3,234)	2	16	48,584	48,681	48,630	0.912	0.00	0.49/0.51
	3	22	47,578	47,711	47,642	0.908	0.00	0.47/0.11/0.42
	**4**	**28**	**46,982**	**47,152**	**47,063**	**0.916**	**0.00**	**0.21/0.30/0.37/0.12**
	5	34	46,387	46,594	46,486	0.902	0.00	0.37/0.11/0.21/0.23/0.08
	6	40	45,874	46,118	45,991	0.876	0.00	0.18/0.26/0.24/0.07/0.16/0.08

CGSS + year represents the corresponding year of the China General Social Survey; N is the sample size; black bolded numbers are the best-fit categories.

The results indicate that in the 2010 data fitting, the six-category model exhibited the best fit indices, including AIC, BIC, and a-BIC, particularly with an Entropy value of 0.937, the highest among all categories. However, category probabilities revealed that one class only accounted for 3%, falling below the minimum threshold of 5%, rendering the six-category model unsuitable. The four-category model also demonstrated good fit indices, with an Entropy value of 0.916 and category probabilities meeting the required criteria, making it the optimal model. In the 2013 data fitting, the four-category model had the best fit indices, with an Entropy value of 0.889, and all category probabilities exceeded 0.05, confirming it as the optimal model. For the 2015 data fitting, while the six-category model had the highest Entropy value of 0.928, one class accounted for only 4%, again below the minimum threshold, leading to the conclusion that the four-category model was more appropriate. In the 2017 data fitting, the two-category model achieved the highest Entropy value of 0.941; however, its AIC, BIC, and a-BIC values were also the highest among all categories. Comparatively, the four-category model had an Entropy value of 0.914, the second highest, and its AIC, BIC, a-BIC values and category probabilities met the required standards. In the 2021 data fitting, the four-category model yielded the highest Entropy value of 0.916, with acceptable category probabilities and AIC, BIC, and a-BIC values of 46,982, 47,152, and 47,063, respectively. Overall, the four-category model is deemed superior.

In summary, all 5 years—2010, 2013, 2015, 2017, and 2021—exhibit four distinct types of gender role attitudes among women, with no notable changes in types over time. Thus, the study selects these four categories as the ideal types and calculates the scores of each type across various indicators of gender role attitudes. By comparing these scores, names are assigned to each type. As shown in Table 2, score differences across indicators are generally consistent across the 5 years, allowing for uniform naming of the types. First, Type 1 scores significantly above average in gender division of labor, gender abilities, and romantic inclinations, with slightly higher scores than other types in labor market competition and household division of labor, making it the most traditional type among all. This type is therefore named “Multidimensional Traditional.” Next, Type 2 scores high in gender division of labor, while the other four indicators are around average, indicating a more traditional view primarily in the area of gender division; thus, it is named “Traditional Division of Labor.” Then, Type 3 scores relatively high in gender abilities, with other indicators around average, suggesting a traditional view mainly regarding innate gender capabilities, and is named “Innate Ability Traditional.” Finally, Type 4 scores lower than other types and below average across all five indicators, showing highly modern gender role attitudes across dimensions, so it is named “Fully Modern.”

Across the multi-point data from 2010 to 2021, the four types of gender role attitudes—Multidimensional Traditional, Traditional Division of Labor, Innate Ability Traditional, and Fully Modern—have consistently been present. Among these, the Multidimensional Traditional and Fully Modern types represent two “polarized” expressions of gender role attitudes. The Multidimensional Traditional type reflects the most conservative segment among Chinese women, with a pronounced male-prioritizing tendency and high dependence on men in gender perceptions. They believe that men are inherently more capable than women, and therefore, men should pursue success in their careers, while a woman’s value is inherently tied to her role within the family. In contrast, the Fully Modern type embodies the “new era of independent women," advocating gender equality in all respects. They strongly oppose traditional gender notions such as male dominance and female submission, asserting that men and women are born equal, each possessing unique strengths and abilities. The Traditional Division of Labor and Innate Ability Traditional types are “contradictory” forms of gender role attitudes. These groups generally demonstrate egalitarian views across most dimensions but retain traditional views specifically in terms of gender division of labor or innate abilities. Essentially, they possess relatively modern gender perspectives, yet certain realities and cognitive limitations lead them to hold on to some traditional attitudes. Thus, gender role attitudes among contemporary Chinese women reveal a complex and diverse profile (Table 3).

**Table 3:** Scores for each of the four types of Chinese women’s gender role attitudes.

Year	Dimension	T1 Multidimensional traditional	T2 Traditional division of labor	T3 Innate ability traditional	T4 Fully modern	Average
2010	A421	**4.42**	**4.18**	2.17	1.81	3.58
	A422	**4.25**	2.01	**3.78**	1.66	2.99
	A423	**3.71**	3.05	3.04	2.50	3.20
	A424	2.46	1.84	2.06	1.55	2.05
	A425	2.11	2.00	1.96	1.85	2.01
2013	A421	**4.20**	**3.99**	2.42	1.86	3.38
	A422	**4.05**	2.02	**3.50**	1.72	2.92
	A423	**3.63**	3.07	3.12	2.46	3.14
	A424	2.54	1.89	2.29	1.66	2.14
	A425	2.27	2.07	2.28	2.03	2.16
2015	A421	4.19	3.89	1.92	1.83	3.28
	A422	4.12	2.19	3.94	1.79	2.97
	A423	3.70	3.03	3.05	2.52	3.16
	A424	2.59	2.05	1.93	1.66	2.15
	A425	2.16	2.12	1.93	2.00	2.09
2017	A421	**4.26**	**4.06**	2.04	1.73	3.12
	A422	**4.15**	2.08	**3.77**	1.69	2.86
	A423	**3.76**	3.13	3.02	2.34	3.07
	A424	2.45	1.96	1.98	1.56	2.00
	A425	2.13	2.09	2.07	1.93	2.05
2021	A421	**4.35**	**4.05**	1.99	1.64	3.00
	A422	**4.26**	1.98	**3.79**	1.57	2.74
	A423	**3.77**	3.00	2.92	2.17	2.92
	A424	2.44	1.80	1.78	1.42	1.85
	A425	2.02	1.94	1.81	1.62	1.82

Black bolded numbers indicate that each type scored much higher than the overall average on this item; A421, A422, A423, A424, and A425 represent the five sub-dimensions of gender role attitudes of gender division of labor, perception of gender competence, marital and relationship tendency, competition in the labor market, and division of household chores, respectively.

### 4.2 Analysis of the mechanisms influencing category differentiation

This study employs the latest 2021 cross-sectional data to analyze the factors influencing the differentiation of gender role attitudes among Chinese women. Based on the posterior probability estimates from the four-category latent profile model, each sample is assigned to one of the four categories. An unordered multinomial logistic regression model is constructed, comparing each type pairwise to examine factors that differentiate the group in question from the other three, which lean more modern (Lubke and Muthén, 2007). First, the model’s fit is tested: the −2 log-likelihood value is 3199.82, with a chi-square value of 1,015 and a *p*-value of 0.000, below the 0.001 threshold. The Pearson chi-square value is 2,201, with a *p*-value of 0.638, exceeding 0.05. Thus, the overall model fit is satisfactory.

Using the Multidimensional Traditional type as a reference group, this analysis examines how factors such as mother’s education, father’s education, marital status, urban–rural residency, age, personal income, political affiliation, employment status, and individual educational level impact the likelihood of women’s gender role attitudes aligning with the other three types. The *p*-values for the Social Division Traditional, Innate Ability Traditional, and Fully Modern types are all below 0.05, indicating that these factors significantly influence differentiation between the Multidimensional Traditional type and the other three categories. Therefore, the final results include three regression models. Through the Odds Ratio (OR), this study further categorizes and analyzes the impact of these 10 factors on the alignment of gender role attitudes with each type (Table 4).

**Table 4:** Results of unordered multicategorical logistic regression.

Variable	T1/T2		T1/T3		T1/T4		T2/T3		T2/T4		T3/T4	
	B	OR	B	OR	B	OR	B	OR	B	OR	B	OR
Mother’s education (none)	−0.00	1.00	0.17	1.19	0.33**	1.39	0.18	1.19	0.33*	1.39	0.15	1.17
Father’s education (none)	0.29*	1.34	0.33*	1.39	0.08	1.09	0.03	1.03	−0.21	0.81	−0.24	0.79
Marital status (married)	0.11	1.12	0.14	1.15	0.76***	2.15	0.03	1.03	0.65***	1.92	0.62***	1.86
Registered residence (rural)	0.03	1.03	0.37*	1.44	0.58***	1.78	0.33*	1.40	0.54***	1.72	0.21	1.23
Age (old)												
Young	0.41*	1.50	0.81***	2.25	1.28***	3.61	0.40*	1.50	0.87***	2.40	0.47*	1.60
Middle	−0.14	0.87	0.20	1.23	0.26	1.29	0.34*	1.41	0.40**	1.49	0.05	1.06
Annual personal income (low)												
High	0.35	1.42	0.62*	1.87	0.62**	1.87	0.27	1.31	0.27	1.31	0.00	1.00
Middle	0.32**	1.37	0.47***	1.60	0.65***	1.91	0.15	1.17	0.33**	1.39	0.17	1.19
Political profile (masses)	0.49*	1.64	0.17	1.19	0.70***	2.01	−0.32	0.73	0.20	1.22	0.52**	1.69
Subjective class identity (low)												
High	−0.34	0.71	−0.63**	0.54	−0.42*	0.66	−0.29	0.75	−0.08	0.92	0.20	1.22
Middle	−0.07	0.93	−0.33*	0.72	−0.12	0.89	−0.25	0.78	−0.05	0.96	0.21	1.23
Employment status (unemployed)												
Non-agricultural work	−0.04	0.96	0.03	1.03	0.11	1.12	0.07	1.07	0.15	1.16	0.09	1.09
Worked in agriculture	−0.23	0.79	−0.36*	0.70	−0.59***	0.56	−0.13	0.88	−0.35*	0.70	−0.23	0.80
Individual’s educational level (junior)												
High	0.60*	1.82	1.26***	3.54	1.52***	4.57	0.66*	1.94	0.92***	2.51	0.26	1.29
Middle	0.50**	1.64	0.76***	2.14	0.98***	2.67	0.27	1.31	0.49***	1.63	0.22	1.25

* *p* < 0.05, ** *p* < 0.01, *** *p* < 0.001; values in parentheses indicate reference categories for independent variables, with the first part before “/” denoting the reference group for dependent variables; T1 = Multidimensional Traditional, T2 = Traditional Division of Labor, T3 = Innate Ability Traditional, T4 = Fully Modern.

Based on the theories of external environment determinism and internal interest determinism, and synthesizing the four types and characteristics of gender role attitudes among Chinese women, this study identifies three mechanisms influencing the differentiation of gender role attitudes.

#### 4.2.1 External environment-driven mechanism

From an external perspective, the family stands as a primary factor shaping different types of gender role attitudes among women. Parental gender role beliefs often directly influence those of the next generation. Parents with educational backgrounds tend to hold more egalitarian views on gender roles, and a family atmosphere of gender equality can encourage children’s gender role attitudes to align with modern values. Marriage also has a significant impact on women: on one hand, it is seen as an important channel for social mobility; on the other, patriarchal culture frequently reinforces traditional gender roles within marriage by placing dual pressures on women through both family and work expectations. Unmarried women, without the constraints of family, often have more freedom to pursue career and personal development, thereby fostering greater recognition of self-worth. Consequently, unmarried women are more likely to belong to the Fully Modern type in terms of gender role attitudes. Additionally, the urban–rural divide, a concentrated expression of societal external factors, greatly influences women’s gender role attitudes. Urban areas, more exposed to modernization, tend to foster a stronger sense of individualism and gender equality among women compared to rural regions. Studies have found that rural women, due to household registration restrictions, are unable to access urban health insurance, thus being forced into an intergenerational caregiving contract, where they exchange caregiving for their grandchildren in return for financial support from their sons (Cai and Huang, 2017). Their traditional gender role attitudes are significantly higher than those of women who work outside the home. Lastly, age differences inherently reflect the distinct ideological shifts of different eras. Older women, shaped by more conservative times and traditional lifestyles, are more likely to hold Multidimensional Traditional attitudes. By contrast, contemporary young women, growing up in the era of the internet and exposed to diverse ideologies, tend to embrace more egalitarian gender roles. Young people are also generally more receptive to progressive ideas and education, making their gender role attitudes more modernized.

#### 4.2.2 Internal interest-driven mechanism

From the perspective of intrinsic benefits, a woman’s personal income serves as a significant internal factor influencing her gender role attitudes. A commendable income empowers women with a certain degree of agency in the dynamics of gender power. Low-income women are more likely to exhibit gender role attitudes that align with the multidimensional traditional type, whereas women with higher incomes are more likely to alleviate some of the household pressure by outsourcing domestic services (Liu and Tong, 2014). Economic independence among women facilitates a transition in their gender role attitudes from traditional to modern. In terms of subjective class, women in higher strata tend to lean toward multidimensional traditional attitudes, while those in lower strata are more inclined toward fully modern or innate capability traditional attitudes. According to the theory of disadvantaged status, as a woman’s class standing rises, her perception of inequality diminishes, making it increasingly difficult for her to develop modern gender role attitudes.

#### 4.2.3 Integrated driving mechanism

Employment status and a woman’s level of education jointly influence her gender role attitudes through the interplay of external environment and intrinsic benefits. Research indicates that women with full-time jobs tend to hold more egalitarian views on gender compared to their unemployed counterparts. In the context of China’s pronounced urban–rural divide, women engaged in agriculture often possess even more traditional gender role attitudes than unemployed women. This may stem from their prolonged residence in rural areas, where the deeply entrenched belief of “men as breadwinners and women as homemakers” significantly impacts their perspectives, alongside their primary involvement in labor-intensive agricultural activities, which exacerbates doubts regarding women’s capabilities. Moreover, women who have long worked in agriculture face persistent obstacles to personal development and empowerment within the confines of a “male labor, female farming” division of labor. The traditional gender division logic of “men outside, women inside” has not only remained unadjusted but has also been “modernized,” rendering it more insidious. Consequently, women in agriculture may reinforce the inequality in gender social status.

Finally, education plays a pivotal role in fostering modern and equitable gender role attitudes. Well-educated women typically exhibit a distribution of gender role attitudes across innate capability traditional, social division traditional, and fully modern categories, all leaning toward modernization. Particularly, women with higher education are significantly more likely to possess fully modern gender role attitudes compared to those with lower educational attainment. On one hand, increased educational levels provide individuals with greater opportunities to embrace values of gender equality, thereby reshaping their own beliefs and behavioral patterns and mitigating the impact of traditional gender roles and stereotypes. On the other hand, higher education yields numerous additional benefits for women, such as increased economic income and enhanced agency.

### 4.3 The evolutionary trends of different types of gender role attitudes

#### 4.3.1 Overview of evolutionary trends

**Figure 1:**
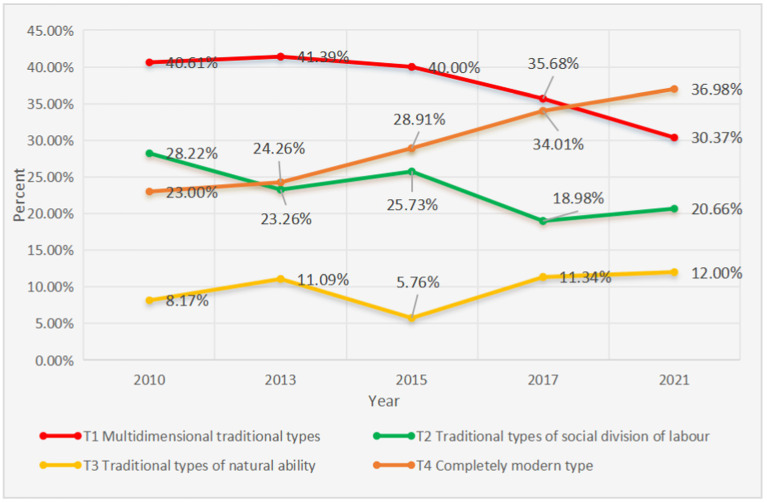
Overall evolution of gender role attitudes among Chinese women (2010–2021). The horizontal axis represents the years, while the vertical axis indicates the proportion of individuals corresponding to the four types of gender role attitudes.

From 2010 to 2021, there has been a significant decline in the proportion of women exhibiting “multidimensional traditional” gender role attitudes, while the share of those embodying the “fully modern” type has markedly increased, rising to prominence in 2021. Overall, since 2010, the overall level of gender role attitudes among Chinese women has been on the rise, indicating a general trend toward modernization in their perspectives (see Figure 1).

**Figure 2:**
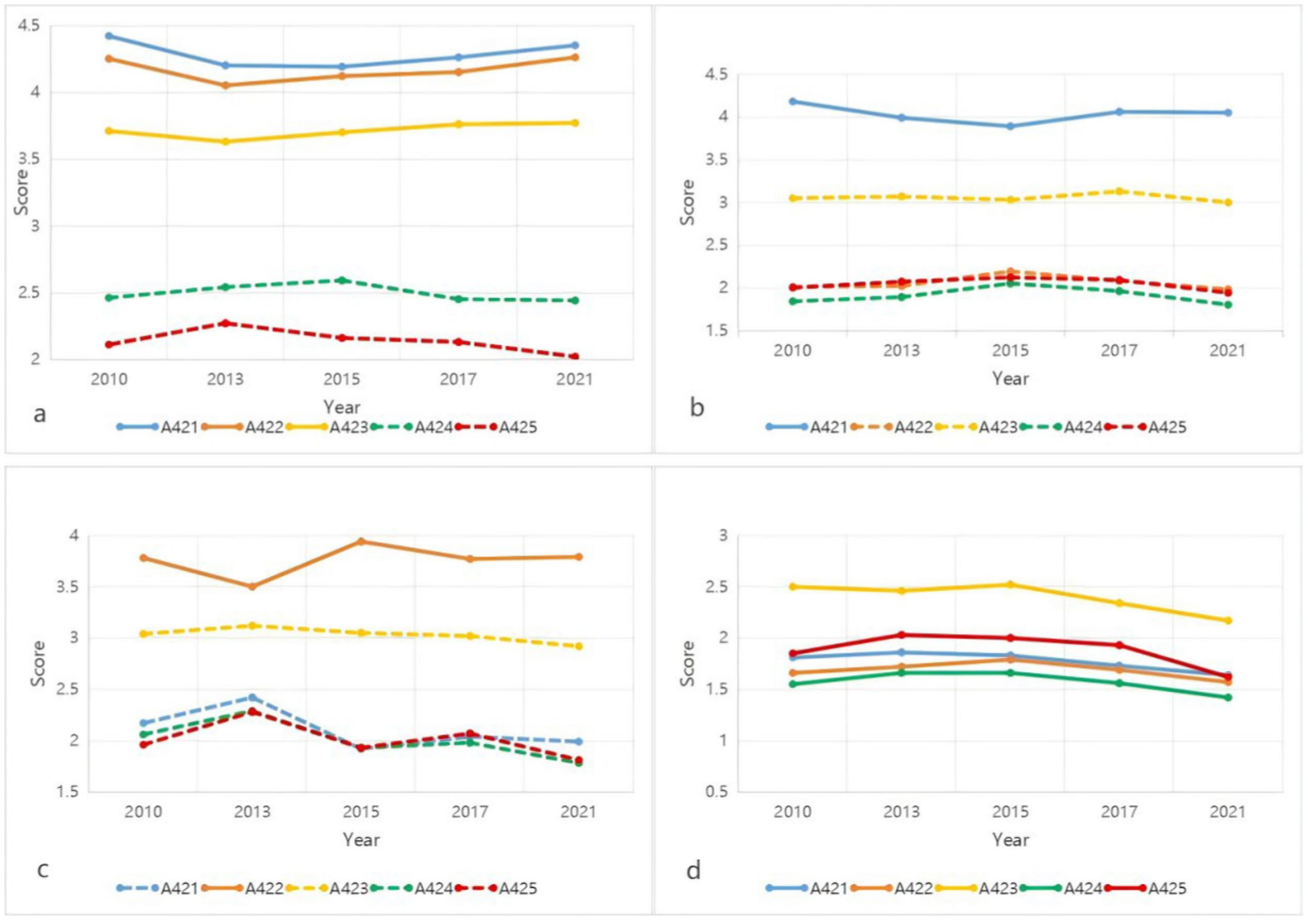
Temporal evolution trends of the four types of gender role attitudes. **(a–d)** Represent the temporal evolution characteristics of gender role attitudes for the Multidimensional Traditional, Traditional Division of Labor, Innate Ability Traditional, and Fully Modern types, respectively.

The differentiation and evolution of gender role attitudes among Chinese women display a diversified trend (see Figure 2). For the “Multidimensional Traditional” type, all three core indicators mark 2013 as a turning point: prior to this, gender role attitudes among Chinese women were progressing toward modernization, but post-2013, they have consistently reverted toward traditional perspectives. The evolution of the “social division traditional” type, particularly concerning the core indicator of gender division, experienced a similar shift around 2015, after which it began to revert toward traditional attitudes, reaching a relatively traditional level by 2021. The “innate capability traditional” type has exhibited a fluctuating state regarding the core indicator of gender capabilities, constantly shifting in its direction of change. As for the “fully modern” type, its indicators demonstrated a slow upward trend prior to 2015, but thereafter, a marked and sustained decline became evident.

In summary, since 2010, the overall trend of gender role attitudes among Chinese women has been toward modernization. However, a precise analysis reveals that the evolutionary trends of different female groups holding various types of gender role attitudes exhibit notable differences. The fully modern type, after a brief regression toward traditional views, has continued on a path toward modernization. In contrast, both the multidimensional traditional type and the social division traditional type have shown signs of slight regression toward traditional attitudes following their development toward modernization. Meanwhile, the innate capability traditional type has remained in a state of fluctuation.

#### 4.3.2 Analysis of the causes of differential evolution

The multidimensional traditional type is the result of the dual pressure from structural risks and traditional rationality. Its dominance in the early stages can be attributed to three structural constraints: the cultural reproduction of Confucian ethics, institutional exclusion, and the absence of social security during the early stages of the market economy. Before 2013, urbanization and the growth of the service sector promoted rural women’s participation in the workforce, contributing to the modernization of gender role attitudes. After 2013, the slowing economic growth combined with the consolidation of the household registration system forced this group to revert to traditional protective strategies. For instance, when household income declined, women’s recognition of the “male breadwinner” role increased, reflecting a logic of risk aversion.

The social division traditional type results from the interplay between policy interventions and cultural habits. After the implementation of the “National Program for the Development of Women in China” in 2010, the strengthening of gender equality in the public sector advanced the modernization of occupational division awareness. After 2015, the comprehensive relaxation of the two-child policy reinforced maternal responsibilities, and combined with Confucian cultural narratives, family division of labor began to regress toward traditional norms.

The innate capability traditional type reflects the ongoing struggle between cultural traditions and educational empowerment. The fluctuations in the “naturally endowed ability traditional type” stem from the contest between traditional pull and modern push: the persistent reproduction of the “male strong, female weak” mindset within the hierarchical structure, while the expansion of higher education mitigates ability biases. During economic downturns, the increase in implicit gender discrimination in corporate recruitment intensifies these fluctuations.

The fully modern type demonstrates the dynamic balance between institutional benefits and market constraints. Before 2015, its slow rise benefited from the gender-neutral labor market and the spread of individualistic values. After 2015, there was a short-term decline due to the surge in employment pressures under the new normal economic conditions, leading some highly educated women to strategically accept traditional marital arrangements. After 2018, the improvement of anti-discrimination laws in employment and the development of the digital economy have driven a continuous increase in the proportion of this group.

## 5 Conclusion and discussion

### 5.1 Research conclusions

The conclusions of this research are as follows.

First, the gender role attitudes of contemporary Chinese women exhibit a diversified characteristic. This study identifies four typical types of gender role attitudes among Chinese women: multidimensional traditional type, social division traditional type, innate capability traditional type, and fully modern type. Women with “multidimensional traditional” gender role attitudes display a highly traditional understanding of gender concepts across multiple dimensions. The “social division traditional” and “innate capability traditional” types represent a conflicting stance situated between traditional and modern views, where gender role attitudes tend to be more egalitarian in most dimensions, only reverting to traditional perspectives in specific areas of gender division or capability. Women embodying the “fully modern” gender role attitudes demonstrate the most comprehensive and profound awakening of self-awareness, exhibiting modern and equitable behavior across all dimensions. Overall, there is a notable divergence in the degree of modernization of gender role attitudes among Chinese women.

Second, this study identifies three categories of mechanisms that lead to the differentiation of gender role attitudes among Chinese women. The first category is externally driven factors, which primarily operate from the outside. Among these, parental education level is a key determinant. Research indicates that women whose parents are well-educated, urban, young, and unmarried are more likely to exhibit “fully modern” gender role attitudes, while rural, elderly, and married women are more inclined to hold “multidimensional traditional” gender role attitudes. The second category encompasses internally driven factors, which primarily operate from within. Women with lower personal income and without political beliefs are more likely to possess “multidimensional traditional” gender role attitudes. Notably, contrary to many research findings, women with a higher subjective class identification tend to lean toward traditional gender role attitudes. The third category involves a combined influence of external and internal factors, which operate through both pathways. Research shows that factors such as lower education levels and engagement in agriculture contribute to a traditional orientation in women’s gender role attitudes.

Third, overall, since 2010, the pattern of gender role attitudes among Chinese women has shifted from a dominance of “multidimensional traditional” types to a predominance of “fully modern” types, indicating a significant awakening of gender equality awareness among Chinese women. However, a precise analysis reveals distinct evolutionary trends among the four groups of women holding different types of gender role attitudes, all of which exhibit varying degrees of regression toward traditional views. Firstly, the core indicators of the “multidimensional traditional” type initially progressed toward modernization, followed by a sustained regression toward traditional attitudes. Similarly, the core indicators of the “social division traditional” type also experienced a progression toward modernization before reverting to traditional views. The core indicators of the “innate capability traditional” type have fluctuated between traditional regression and modernization without a clear trajectory. In contrast, the indicators of the “fully modern” type are predominantly modern, showing a brief regression toward traditional views before rapidly advancing toward modernization.

### 5.2 Research discussion

Gender equality signifies the parity of roles between men and women, which is crucial for the progress and development of a nation. Although significant advancements have been made in the global pursuit of gender equality, and people’s gender role attitudes are gradually modernizing, traditional patriarchal influences continue to permeate societal views on gender. Gender inequality persists across various classes, cultural backgrounds, ethnicities, and regions. Many countries uphold cultural customs and norms that exhibit a “male preference,” with parents often more inclined to invest in the human capital of sons rather than daughters (Jayachandran, 2015). Traditional gender divisions, such as “men as breadwinners and women as homemakers,” create a divide that harms both genders, contributing to the current imbalance in societal gender ratios. The pervasive nature of gender discrimination not only hinders women’s growth but also impedes the future of nations. The advancement of social civilization should ideally provide greater developmental opportunities for both genders, ensuring that both men and women possess the right to realize their self-worth. To sustain the momentum of social progress, it is essential to continually focus on issues of gender equality and strive to advance the cause of gender parity.

This study makes several significant contributions to the research field. Firstly, previous studies have often regarded gender role attitudes as an indivisible whole (Liu and Tong, 2014). However, this research innovatively identifies four distinct types of gender role attitudes among Chinese women through latent profile analysis. This enriches the theoretical study of gender role attitudes. Furthermore, this study introduces the latent profile analysis method into research on gender role attitudes. This classification approach avoids the bias caused by arbitrarily assigning the number of categories, marking a significant methodological advancement. Secondly, building upon existing research (Xu et al., 2010), the article further analyzes the mechanisms through which various factors influence the differentiation of gender role attitudes among Chinese women, based on the characteristics of the four identified types. This has practical implications for addressing the social issue of gender inequality. Lastly, this paper accurately delineates the evolutionary trends of gender role attitudes among Chinese women from multiple perspectives, thereby providing an important complement to previous studies (Thornton and Young-DeMarco, 2001; Valentova, 2013; Wu et al., 2022). This study comprehensively reveals the evolutionary trends of the four types of gender role attitudes among Chinese women since 2010. This study reveals significant differences in the characteristics and evolution trends of various types of gender role attitudes among Chinese women. Particularly, women with a multidimensional traditional gender role attitude continue to exhibit traditional views across many dimensions of gender role awareness, which does not fully align with the fundamental principles of modernity theory. The notion of gender equality in developing countries, represented by China, is heterogeneous, marking an important breakthrough in modernity theory.

The limitations of this study and prospects for future research are as follows. Firstly, this study identifies different types of gender role attitudes through quantitative analysis, but lacks depth in examining the influencing factors of type differentiation. Future research should incorporate qualitative methods such as interviews to further explore the formation mechanisms of various gender role attitudes. Secondly, the study does not include male samples. Since the research primarily addresses the gender role attitudes of Chinese women, only female samples were selected, leaving the gender role attitudes of men unexamined. This limitation prevents the formation of comparative conclusions between the two genders. Third, the multinomial logistic regression analysis offers advantages in examining categorical variables and multiple complex factors. This study employs this method to explore the influencing factors of the differentiation of women’s gender role attitudes. However, this approach has limitations in incorporating more control variables and revealing interactions between independent variables, suggesting the need for new data analysis methods in future research. Fourth, the data for this study is derived from the CGSS. While this source is highly representative and authoritative, the data is still subject to issues such as cross-sectional data and self-reporting bias.

## Data Availability

The datasets presented in this study can be found in online repositories. The names of the repository/repositories and accession number(s) can be found at: http://doi.org/10.6084/m9.figshare.27642888.
